# Green transformational leadership and green creativity? The mediating role of green thinking and green organizational identity in SMEs

**DOI:** 10.3389/fpsyg.2022.977998

**Published:** 2022-09-21

**Authors:** Basheer M. Al-Ghazali, Hamid Mahmood Gelaidan, Syed Haider Ali Shah, Rafia Amjad

**Affiliations:** ^1^Interdisciplinary Research Center for Finance and Digital Economy (IRC-FDE), Dammam Community College, King Fahd University of Petroleum and Minerals, Dhahran, Saudi Arabia; ^2^Department of Management and Marketing, College of Business and Economics, Qatar University, Doha, Qatar; ^3^Business Studies Department, Bahria University, Islamabad, Pakistan; ^4^Management Sciences Department, Bahria University, Islamabad, Pakistan

**Keywords:** green transformational leadership, green creativity, green thinking, green organizational identity, small and medium sized enterprises (SMEs)

## Abstract

For countries and organizations to achieve sustainable development, radical green creativity is required. Despite the fact that the influencing elements of green creativity have received a lot of attention, there is little research on the antecedents of green creativity. The current study attempted to fill the gap by exploring the underlying mechanism of green thinking and green organizational identity as mediators. This study aimed to examine the impact of green transformational leadership on green creativity through mediators, such as green thinking and creative organizational identity in SMEs. We gathered data from 460 respondents from SMEs operating in Pakistan using a survey questionnaire. The findings revealed that green transformational leadership had a significant impact on green organizational identity, which cultivated green creativity in SMEs. Additionally, results portrayed that green organizational identity performed mediation in the relationship between green transformational leadership and green creativity. Moreover, green thinking mediates the association between green transformational leadership and green creativity. This study offers novel insights into how to stimulate green transformational leadership and cognitive processes in SMEs to encourage green creativity. The implications for management and practitioners are discussed in light of the study's findings.

## Introduction

Sustainable green management concepts and practices have recently acquired interest among businesses and academics as a way to minimize the harmful effect of industrial waste and pollution (Li et al., 2020). Stakeholders and academics have urged organizations to develop policies that will achieve social, economic, and environmental goals. The concept of sustainability follows Lopez-Cabrales and DeNisi (2021), and can be defined as “an attempt to strike a balance between the economic, social, and environmental goals of companies,” and contextualize green leadership as a dimension of sustainability, while earlier green business models focused mostly on enhancing environmental process performance and asset utilization. However, newer models concentrate on growth planning, manufacturing methods, and pre- and post-design implications, resulting in enhanced long-term viability (Begum et al., 2022). Recent research studies have suggested that greening SMEs can assist businesses in lowering carbon pollution, waste storage, and power consumption (Bai et al., 2017; Awan et al., 2019; Ojo et al., 2019). As a result, the focus of research in academic and industrial fields is shifting from a broad discussion to a specific topic, which is of greening certain functional departments, green finance (Przychodzen et al., 2018), such as green innovation and green creativity (Zailani et al., 2015; Awan et al., 2019), and green human resource management and the practices (Yong et al., 2019; Shah et al., 2020).

Various scholars have argued that businesses that take steps toward green processes, green creativity, and innovation will benefit from being first-movers in a field with production that is both environmentally friendly and resource-efficient, which will increase their corporate reputation and share of the market (Packalen, 2010; Yong et al., 2019; Li et al., 2020). According to these researchers, businesses may turn green creativity into environmentally friendly products and services to help the environment (Jia et al., 2018; Eide et al., 2020; Li et al., 2020). Furthermore, by integrating green creativity into the firm's major concerns, firms can acquire a green competitive advantage over their competitors in such a volatile environment (Chen, 2008; Jia et al., 2018).

The development of novel and valuable green ideas about producing green products, methods and practices, or delivery is referred to as green creativity (Chen and Chang, 2013; Song and Yu, 2017; Li et al., 2020). Moreover, the ability to come up with creative, novel, and worthwhile ideas is referred to as creativity (Wyer et al., 2010). It is determined by a variety of organizational and individual factors. A study conducted by Chen and Chang (2013) showed that leadership and organizational attitudes around environmental concerns influence green creative thinking. Green transformational leadership (GTL) encourages employees to engage in exhibiting green behavior, which ultimately cultivates the behavior of concern about the environment in a way that they may care for water and paper utilization in an effective manner which later can be reutilized and remain environmentally friendly (Mittal and Dhar, 2016; Li et al., 2020). The following empirical research supports the argument that green leadership and other factors play a role in developing green creative behavior (Mittal and Dhar, 2015; Tuan, 2019; Singh et al., 2020). Although green transformative leadership is a critical component of any firm to support green creativity (Li et al., 2020; Singh et al., 2020), the relationship between green transformational leadership and an environmentally integrated development practice and management system has been studied by a number of researchers (Chen, 2011; Garg and Dhar, 2014; Singh et al., 2020). GTL, according to these researchers, has an indirect impact on environmental-related behavior and performance, while in some studies, it is found to have a moderated role and mediating role (Li et al., 2020; Singh et al., 2020). However, there is still a need to come up with empirical evidence of a direct link between green transformational leadership and environmental performance. The impact of green transformational leadership on employee motivation, as well as the indirect effects of GTL on organizational environmental performance, such as lower energy usage and enhanced recycling capabilities, has been studied (Chen, 2011; Mittal and Dhar, 2016; Li et al., 2020).

The role of transformational leaders is very vital in creating and building the vision that leads to proactive steps toward the different tasks and achieving environmental concerns and green initiatives (Bass, 2000; Sun et al., 2022). In addition, transformational leaders support the culture of innovative ideas, implementation of those ideas in terms of actions, and building the model of “creativity-enhancing forces” (Sun et al., 2022). The role of TL is immensely important in the success and implementation of innovative business ideas (Elkins and Keller, 2003; Rehman et al., 2021; Sun et al., 2022). With the same notion, the role of TL in promoting a sustainable environment is also very crucial but still less focused worldwide, particularly in a developing country like Pakistan (Sun et al., 2022). A study conducted in the Pakistani context found a positive and significant impact of TL on green performance (Zafar et al., 2017).

Similarly, Pakistan being a developing country faces environmental threats and issues due to climatic change and global warming as narrated in the IQAir (2021), and a province of Pakistan (Lahore) is found to be the second on the list of highly polluted cities globally. The focus of SMEs has shifted to environmental degradation (Sun et al., 2022 reference). Moreover, environmental concerns and issues have paved the way for practicing green activities, particularly green creativity (Chen, 2011; Sun et al., 2022). Since the majority of the SMEs are located in the Punjab Province, this study was mainly conducted in the Punjab Province of Pakistan (Shah et al., 2021; Sun et al., 2022). A quite number of studies have been conducted to examine the impact of TL on environmental performance and other factors (Sun et al., 2022). However, the influence of the TL on green creativity has received less attention (Li et al., 2020; Sun et al., 2022), particularly in the SMEs which are the backbone of the Pakistan economy (Shah et al., 2021). In addition, very few studies have been conducted to investigate the mediating role of green organizational identity and green thinking.

Transformational leadership has been reported to exert a significant impact on fostering employees' creativity in previous studies (Gumusluoglu and Ilsev, 2009; Mittal and Dhar, 2015, 2016; Salas-Vallina and Alegre, 2018; Caldera et al., 2019). Hence, the purpose of this research is to examine the relationship between GTL and green creativity in a business scenario, such as SMEs, from an environmental or green standpoint. GOI was found to be critical in cultivating green creativity in employees working in the hospitality industry (Sethi, 2000; Garg and Dhar, 2014). Employee behavior is influenced by organizational identity, which provides a framework of reference for managers to clarify strategic issues (Fiol, 1991; Garg and Dhar, 2014). Subsequently, organizational identity is a crucial element in molding the employees toward environmental concerns (Chen, 2011; Li et al., 2020). Environmental leaders create a GOI, and inspire them to think, act, and identify themselves toward pro-environmental behavior and organizational efforts toward the environment (Chen, 2011; Mittal and Dhar, 2016). As a consequence, the employees' creative activities are enhanced, and they have the possibility to engage in creative and better performance (Fiol, 1991; Benet-Martínez et al., 2002; Mittal and Dhar, 2015; Kaltiainen and Hakanen, 2022), another gap this research study tries to fill by investigating the role of GTL in shaping green organizational identity and its indirect effect on green creativity in the workplace of SMEs. In addition, green thinking encourages environmentally friendly business practices, such as the use of sustainable materials and energy-efficient manufacturing methods to lower carbon emissions, and result in better environmental practices (Caldera et al., 2019). Green thinkers are environmentally conscious, and they demonstrate their concern for the environment by gardening, planting, and purchasing green products (Ali et al., 2020). Individuals' ability of thinking and feeling about firm greening motivates them to adopt environmentally friendly activities. Green thinking in the workplace enables businesses to create green creativity through green goods, processes, and technologies (Begum et al., 2022). Green transformational leadership influences followers' attitudes, ideals, and thinking by defining to them the green perspective of the world and green methods to incite green production (Ahmeda et al., 2020). As a result, this study anticipates the role of green transformational leadership in developing a sense of the environment's goals, which spark green thinking and encourage employees to participate in green creativity, another gap this study filled by identifying the underlying mechanism of mediation of green thinking between the green transformational leadership and green creativity.

**Figure 1:**
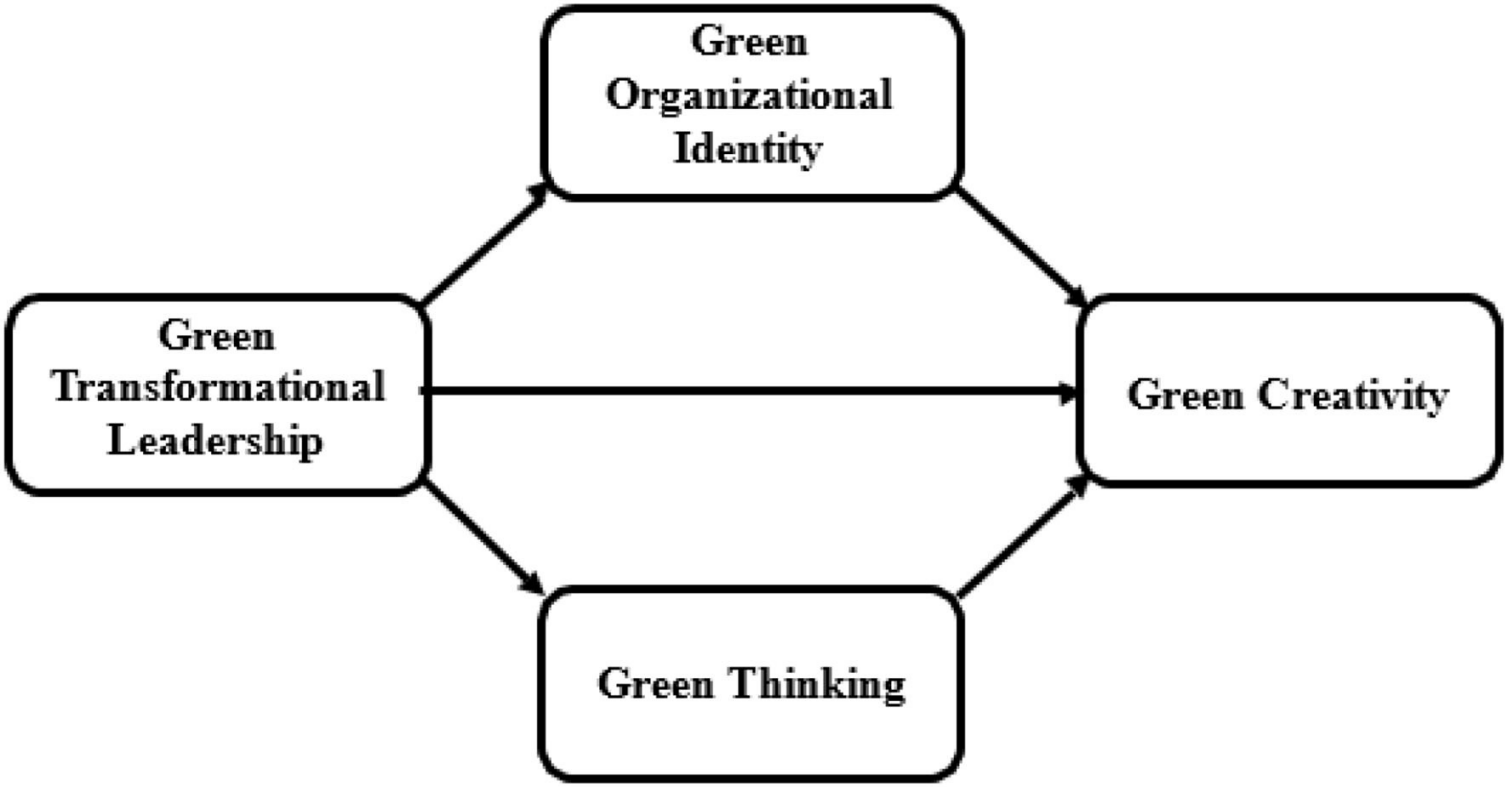
Conceptual framework of the study.

Hence, this research contributes to the current literature in a number of ways: First, it establishes the link between GTL and green creativity. Second, the importance of green organizational identity in mediating the relationship between GTL and green creativity has been explored. Third, the role of green thinking in mediating the relationship between GTL and green creativity has also been investigated for an in-depth understanding of green creativity in SMEs. Hence, the aim of this research is to better understand the function of GTL and green creativity in SMEs in the developing context as mentioned in Figure 1.

## Theoretical foundation and hypotheses formulation

The framework is developed based on Ability Motivation Opportunity (AMO) theory and Resource-based view (RBV) theory. The concept of human capital and its relations to creativity is closely linked and evident in the HRM literature (Takeuchi et al., 2007). The RBV proposed that the firm market position, image, goodwill, and competitive edge are based on strategic resources which are hard to replicate and imperative (Sun et al., 2022). Furthermore, the AMO theory posits that to build the HPWS, three main aspects are immensely important to create a set of HR practices. These three aspects are ability, motivation, and opportunity (AMO) (Appelbaum et al., 2000). Additionally, the ability to build and utilize multiple methods to perform a number of tasks and duties with relevant knowledge and skills can be developed by adopting different training and development, as well as recruitment and selection, programs.

With the same notion, goals are achieved when the staff is motivated through the financial, non-financial, and proper evaluation process (Sun et al., 2022). Lastly, the opportunity reflects the concept of personal liberty, participation in decision-making, involvement, and free flow of information with staff, which are engraved in the set of policies of the organization. Moreover, in building the green training capacity of employees, cooperation from suppliers and customers is also integral (Yu et al., 2020; Ding et al., 2022; Sun et al., 2022). Similarly, the TL and employees' relationship are vital resources of the firm, and the main goal of the firm's GHRM is to create and provide opportunities for development along with the proper motivation, which could lead them to provide a competitive edge to the firm and elevate the performance to the next level (Boxall and Steeneveld, 1999; Sun et al., 2022). Hence, this study foundation is based on the basic standard of RBV that creates the competitive edge and market position of firms through the human resources. Further, The firm's structure and processes are highly connected to the human capital that actually translates the policies and practices into the implementation and results which enable the firm to win over its rivals (Singh et al., 2020; Khan et al., 2021; Sun et al., 2022).

### Green transformational leadership and green creativity

Leaders and their traits have a big impact on organizational creativity (Halbesleben et al., 2003; Lu et al., 2022). because they are at the forefront of improving environmental performance (Andriopoulos, 2001). A thorough literature search showed that a number of studies have advocated and produced evidence to show that transformative leadership has a significant influence on creativity (Woodman et al., 1993; Mittal and Dhar, 2015, 2016). There are four dimensions to transformational leadership: intellectual stimulation, individualized consideration, charisma, and inspirational motivation (Bass, 1985a,b). The charismatic power of the transformational leader promotes the formation of inspirational ideas among the followers, resulting in their respect and hence their loyalty. Individualized consideration enables the transformational leader in instilling a sense of connection in their followers, which leads to the development of mutual concern. Furthermore, with the support of inspirational motivation, the transformational leader not only provides a clear direction to the organization, but also explains the road by which the vision can be accomplished. The transformational leader's intellectual stimulation capability allows them to inspire their followers' cognitive capacities, which leads to the growth and development of followers' creativities capabilities (Avolio et al., 1999; Gong et al., 2009; Mittal and Dhar, 2016).

Those are the leaders who think out of the box and walk the road less seen and driven and take firm actions and initiatives (Shin and Zhou, 2003; Gong et al., 2009). As a result, past research has found a strong link between transformative leadership and workforce innovation (Shin and Zhou, 2003; Mittal and Dhar, 2015). Transformational leaders develop employee creativity by encouraging, supporting, extending a helping hand, and building their confidence to take new initiatives (Jung et al., 2003; Sarros et al., 2008). In literature, it is often found that various studies highlighted that transformational leaders delegate the power and empower the employees to work on their novel ideas and to be more effective in the organization (Jung et al., 2003). In this study, the concept of green transformational leadership has been taken with the same notion as advocated by Bass (1998), Gardner and Avolio (1998), and Chen and Chang (2013) which reiterate that leaders must encourage pro-environmental behavior in employees by inculcating the green behavioral goals and concern for the environment while exhibiting the work in the organization. Moreover, another study highlighted that in the electronics sector, green transformational leadership is linked to green creativity. Based on the above arguments, this study hypothesizes that GTL is linked to green creativity (GC) in SMEs.

**Hypothesis 1**. Green transformational leadership positively relates to green creativity.

### Mediating role of green organizational identity

Organizational identity has been defined by Albert and Whetten (1985) as the “central, distinctive, and enduring characteristic of an organization,” and it comprises three main elements: “ideational, definitional, and phenomenological” (Whetten, 2006, p. 269). Employee behavior is influenced by organizational identity, which provides a frame of reference for managers to clarify strategic issues (Fiol, 1991). Studies have highlighted that it is the organizational identity (OI) that transforms and shapes the employees (Chen, 2011; this paper reference). This study focused on the importance of organizational identity in SMEs from an environmental viewpoint while considering the organizational identity in terms of green (OI).

Many organizational experts have indicated a willingness to investigate the link between leadership and employee creativity (Gumusluoglu and Ilsev, 2009; Gupta et al., 2012). Green transformational leadership was also shown to have a significant impact on green creativity (Liu et al., 2012; Chen and Chang, 2013). Environmental leaders create a (GOI) and persuade employees to identify with, believe in, and consider well about the company (Chen, 2011). As a result, the employees' creative activities are enhanced, and they have the possibility to engage in creative outcomes (Benet-Martínez et al., 2002; Fernandez et al., 2003). Employees are motivated by the organization's green identity to build meaningful interactions with clients and to come up with innovative solutions to problems (Sethi, 2000; This paper reference). The literature studies have also emphasized the significance of GTL in promoting green creativity in the service and many sectors (Mittal and Dhar, 2015, 2016). Therefore, it is hypothesized that as an organization chooses GTL, its GOI grows, which further leads to an increase in GC. The following hypotheses have been developed.

**Hypothesis 2**. Green transformational leadership positively relates to green organizational identity.
**Hypothesis 4**. Green organizational identity positively relates to green creativity.
**Hypothesis 6**. Green organizational identity mediates the relationship between green transformational leadership and green creativity.

### Green thinking and green creativity

Green thinking encourages environmentally friendly business practices, such as the use of sustainable materials and energy-efficient manufacturing methods, to lower carbon emissions and enhance pro-environmental conduct and performance (Caldera et al., 2019). Employees who are green thinkers are environmentally conscious, and they demonstrate their concern for the environment by cultivating, producing, and purchasing green products (Ali et al., 2020). Individuals' ability to think and feel about corporate greening motivates them to adopt environmentally friendly activities. To put it another way, when people are motivated, they will design green structures, green processes, and green products, even if they are more expensive, to protect the environment (Rademaker and Royne, 2018). Individuals have become more conscious of the importance of protecting their communities and the environment, putting pressure on businesses to follow environmental concerns, regulations, and policies. Similarly, the guided principles of any organization are the beliefs and values which engrave the underlying statements of do not cause harm to the environment, which provides the foundation for the green thinking of employees (Caldera et al., 2019). Individuals' green analytical thinking is required to look and consider the environmental concerns in decision-making, designing a product by gaining a thorough grasp of its components, such as decreasing practices that are harmful to the environment, re-designing existing items, and making the products on a more environmentally friendly basis (Xie et al., 2019). Firms must also expand and diversify their technological capabilities, allowing them to move forward with green process innovation and the development of green products (Cristina De Stefano et al., 2016; Begum et al., 2022).

As highlighted by the study, green thinking is vital to minimize pollution and waste in SMEs (Hines, 2009; Verrier et al., 2014; Caldera et al., 2019; Ohno and Bodek, 2019). Green thinking is an approach whose main objective is to utilize less amount of resources and produce more (Caldera et al., 2019). This green thinking leads to creativity and enables the employees to devise activities in multiple ways, such as minimizing time duration, improving overall efficiency, and minimizing the carbon footprint, to sustain in the highly competitive market (Verrier et al., 2014; Thanki et al., 2016). Green thinking and practices explore opportunities to promote green tools and green performance (Verrier et al., 2014). Various studies have highlighted that green practices are positively related to environmental performance (Amani et al., 2015). A green thinking approach promotes the green creativity of the employees toward different activities, such as improving efficiency, enhancing the production process through bottom-line savings, and achieving economic sustainability (Pham et al., 2008; Dhingra et al., 2014; Caldera et al., 2018, 2019).

Based on the above discussion, the following hypothesis has been developed.

**Hypothesis 5:** Green thinking positively relates to green creativity.

### The mediating role of green thinking

For businesses to establish, pursue, and implement a green vision, the role of green leadership is crucial (Lusiani et al., 2020). When it comes to doing business, organizational greening is unquestionably at the top of GTL's priority list (Mittal and Dhar, 2016). The GTL structure creates green teams and provides them with the direction, assistance, and inspiration they need to be dedicated to the business's environmental goals (Wang et al., 2018). Leaders with green concerns and a clear green vision are able to influence their followers' attitudes, beliefs, and thinking in order to instill a strong belief in their ability to accomplish it. GTL gives significance to green projects and their ensuing repercussions to develop a sustainable future through intellectual stimulation, charisma, and expressive character (Li et al., 2020; Begum et al., 2022). Green product development initiatives are more likely in companies with GTL (Chen and Chang, 2013). In the effective implementation of green creativity and innovation, the underlying mechanism of mediation was found to be significant. The relevance of mediating mechanisms for the successful transformation of processes and procedures to achieve green innovation has been emphasized in numerous studies (Ahmeda et al., 2020). The unique impact of GTL propagates the green philosophy of management and motivates employees to consider and care for environmental issues and develop innovative approaches and efforts to modify existing products and manufacturing ways (Singh et al., 2020). Businesses have rethought the products they manufacture and the processes they use to develop them as a result of green thinking (Begum et al., 2022). Employees' ability to think green and care about the environment benefits them in developing and coming up with products that are more environmentally friendly (Ali et al., 2020). Studies highlighted that GTL affects the employees' norms, values, belief system, and their version of a green world and making products with regard to green benefits and green creativity (Ahmeda et al., 2020). As a result, we anticipate GTL instilling a sense of green aims and objectives that spark green thinking and encourage employees to participate in green creativity. Based on the above discussion, the following hypotheses are proposed:

**Hypothesis 3**. Green transformational leadership positively relates to green thinking.
**Hypothesis 7**. Green thinking mediates the relationship between green transformational leadership and green creativity.

## Research method

In this study, the unit of analysis is middle-level managers working in SMEs. In Pakistan's Punjab Province, more than 66% of SMEs are active. According to SEMDA (2020), there were 15,833 SMEs registered in Punjab. There are five industries in total (Textile, Leather/Footwear, Sports, Metal, and Wood and Furniture), and 310 enterprises were chosen, and this study used a cluster sampling technique to collect data from 460 middle-level managers (Bhutta et al., 2009). The five industries listed above were grouped together to form clusters. A total of 780 questionnaires were distributed in each cluster according to their proportion in the total population.

### Data collection instruments

In this study, GTL was measured through a six-item scale adopted from the study of Chen and Chang (2013). To measure green thinking, this study used a four-item scale adopted from Lee (2008), while to measure green creativity (GC), the six-item scale of Chen and Chang (2013) was used. To assess green organizational identity (GOI), the six-item scale adopted from Chen (2011) was used.

## Analysis and results

Before data can be processed, it must first be prepared for analysis. The first step in achieving a normal data distribution is to detect and solve missing values. The imputation method was created, and the mean substitution approach was used (Hair et al., 2014). In addition, kurtosis and skewness measurements were employed and values were found to be 2.315 and 1.258, respectively, and the dataset had a normal distribution (Kline, 2011). Harman single-factor test was used to check for common method biases. The characteristic root of the common factor with the greatest explanatory power is 10.315, which explains 41.325 of the total variation in the absence of factor rotation, according to this study. The majority of covariance between independent and dependent variables is not explained by a single factor. It demonstrates that this study has no major common method bias. Moreover, 63% were men, and 37% of the respondents were women. Table 1 shows the correlation, standard deviation, Cronbach's alpha, and mean values.

**Table 1:** Mean, standard deviation, and intercorrelations.

**S. No**	**All variables**	**Mean (SD)**	**1**	**2**	**3**	**4**
1	Perceived CSR	3.51 (0.87)	**(0.855)**			
2	Ethical leadership	3.65 (0.65)	0.240^*^	**(0.864)**		
3	Moral reflectiveness	3.95 (0.93)	0.557^**^	0.135^*^	**(0.843)**	
4	Pro-environmental behaviors	3.66 (0.81)	0.656^*^	0.450^**^	0.569^*^	**(0.934)**

** Correlation is significant at the 0.01 level (two-tailed).

* Correlation is significant at the 0.05 level (two-tailed). Values in bold are Cronbach's alpha values. SD, Standard deviation.

### Measurement model

To check the convergent and discriminant validity, the CFA series was used. The goodness of fit for all variables was determined using SPSS AMOS 24. When compared to the three-factor, two-factor, and one-factor models, where all the components loaded on a single factor, the four-factor model (GTL, GOI, GT, and GC) was shown to be a superior fit to the data (see Table 2). All the factor loadings were found to be significant at the 0.001 level (Hair et al., 2010, see **Table 4**). Furthermore, this study employed Cronbach's alpha to validate the outstanding dependability of all constructs that were determined to be above 0.70 (see Table 3). Tables 3, 4 demonstrate that the (AVE) values were >0.50. This adds to the convergent validity of the proposed model (Hair et al., 2010). The discriminant validity was established and the values are given in Table 4. As a result, construct validity was established. Moreover, to check for multicollinearity, the VIF was utilized, and it varied from 1.75 to 4.32 (<10), showing that there were no problems with multicollinearity.

**Table 2:** Results of model comparisons using a CFA approach.

**Model**	**λ^2^**	**df**	**TLI**	**CFI**	**IFI**	**NFI**	**RMSEA**	**SRMR**
Four-Factor model (MO)	541.352	274	0.941	0.966	0.969	0.865	0.057	0.0499
Three-Factor model (M1)	149.458	65	0.939	0.936	0.961	0.957	0.060	0.0370
Two-Factor model (M2)	65.615	33	0.963	0.965	0.988	0.965	0.056	0.0390
One-Factor model (M3)	121.995	8	0.812	0.872	0.871	0.887	0.267	0.0592

**Table 3:** Construct validity.

**Construct**	**Number of dimensions**	**Factor loading**	**AVE**	**CR**	**Cronbach's alpha**
Green transformational leadership (GTI)	GTI 1	0.78	0.57	0.85	0.81
	GTI 2	0.81			
	GTI 3	0.78			
	GTI 4	0.75			
	GTI 5	0.89			
	GTI 6	0.77			
Green organizational identity (GOI)	GOI 1	0.78	0.54	0.85	0.87
	GOI 2	0.73			
	GOI 3	0.85			
	GOI 4	0.87			
	GOI 5	0.79			
	GOI 6	0.71			
Green thinking (GT)	GT 1	0.85	0.67	0.67	0.83
	GT 2	0.89			
	GT 3	0.74			
	GT 4	0.81			
Green creativity (GC)	GC 1	0.67	0.55	0.92	0.91
	GC 2	0.75			
	GC 3	0.78			
	GC 4	0.80			
	GC 5	0.62			
	GC 6	0.67			

**Table 4:** Discriminant validity.

**Constructs**	**CR**	**AVE**	**MSV**	**MaxR(H)**	**PCSR**	**PEB**	**CoWkr**	**OI**
GTI	0.854	0.571	0.380	0.911	0.772			
GC	0.912	0.558	0.461	0.920	0.532	0.685		
GT	0.835	0.674	0.495	0.863	0.620	0.678	0.812	
GOI	0.857	0.541	0.493	0.896	0.515	0.670	0.712	0.793

GTI, Green transformational leadership; GOI, Green organizational identity; GT, Green thinking; GC, Green creativity; AVE, Average variance extracted; MSV, Maximum shared variance; MaxR(H), McDonald construct reliability.

Green Transformational Leadership (GTI); Green Organizational Identity (GOI); Green Thinking (GT); Green Creativity (GC); AVE, average variance extracted; MSV, maximum shared variance; MaxR(H), McDonald Construct Reliability.

### Structural model

The proposed model was evaluated to multiple satisfactory goodness of fit measures (Hair et al., 2010) [χ^2^ = 218.741, *df* = 110, χ^2^/*df* = 1.654, (RMSEA) = 0.050, (GFI) = 0.941, (AGFI) = 0.890, (NFI) = 0.940, (RFI) = 0.921, (IFI) = 0.974, (TLI) = 0.964, (CFI) = 0.960]. H1, H2, H3, H4, and H5 were supported, and a statistically significant impact of GTI on GC, GOI, and GT was observed (see Table 5). The conceptual model also showed that two mediators, green organizational identity (GOI) and green thinking (GT), will have a large and positive impact on employees' green creativity (GC). Table 6 shows the standardized estimates and their 95% confidence intervals, which were calculated using 5,000 bootstrapped samples (Jose, 2013). The results of standardized indirect value estimations revealed that green organizational identity mediated considerable mediation between GTL and GC among employees, thus proving H6. Furthermore, the statistical significance of the second mediating relationship of green thinking was demonstrated, thus validating H7.

**Table 5:** Regression results of the structural model and hypotheses test outcomes.

**Hypothesis**	**Predicted relationship**	**Standard path loadings**	**Standard error**	***t*-value**	***P*-value**	**Decision**
H1	Perceived GTL 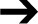 GC	0.53	0.79	6.802	0.001	Supported
H2	Perceived GTL 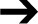 GOI	0.58	0.84	6.987	0.002	Supported
H3	Perceived GTL 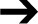 GT	0.77	0.07	7.547	0.004	Supported
H4	GOI 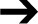 GC	0.58	0.62	6.852	0.002	Supported
H5	GT 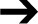 GC	0.56	0.07	6.741	0.002	Supported

GTI, Green transformational leadership; GOI, Green organizational identity; GT, Green thinking; GC, Green creativity.

**Table 6:** Standardized mediation effects: parameter estimate and bootstrap percentile method confidence intervals.

**Hypothesis**	**Parameter**	**Estimate**	**Lower bound**	**Upper bound**	***p*-value**	**Decision**
H6	Panel Ia Perceived GTL 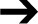 GOI 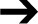 GC	0.344	0.264	0.420	0.014	Supported
H7	Panel IIb Perceived GTL 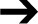 GT 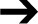 GC	0.275	0.187	0.362	0.011	Supported

GTI, Green transformational leadership; GOI, Green organizational identity; GT, Green thinking; GC, Green creativity.

a) Goodness of fit: χ^2^/df = 1.789, RMSEA = 0.053, GFI = 0.932, CFI = 0.941.

(“GTL 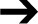 GOI” was constrained to be zero).

b) Goodness of fit: χ^2^/df = 2.225, RMSEA = 0.052, GFI = 0.934, CFI = 0.960.

(“GTL 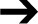 GT 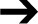
” was constrained to be zero).

## Discussion and conclusion

This research has produced various implications that may be valuable not only for the development of theories, but also offers value for managerial purposes. This is one of the first studies to investigate the impact of GTL and GOI on GC among SMEs in Pakistan. Employees' perceptions of their leader's transformative approaches to directing people to improve GOI, as well as their ideas about their abilities to function creatively, produce a significant impact on their creative performance, according to the findings of this research study. Moreover, green transformative leaders in the hotel industry have been found to appreciate and motivate GC in employees (Gebauer, 2011; Wang et al., 2014; Mittal and Dhar, 2015, 2016). GOI functions as a mediator between GTL and GC, according to the study's findings. Furthermore, as the results exhibit, it is concluded that GTL is linked to GC. As a result, managers at SMEs should work on strengthening their leadership style in order to foster more green creativity. Previous research has revealed that industrial companies, such as the electric sector and hotel industry, have placed a high priority on establishing green transformational leadership in order to boost green creativity (Chen and Chang, 2013; Mittal and Dhar, 2016). Hence, the presence of GTL provides the direction for environmental performance and its implications, which create an impact on the performance of employees' activities, and they come up with creativity while concerning the environment. Moreover, the role of GT and GOI is also crucial to further enhance the GC, which is now the ultimate concern of many organizations. In addition, environmentally friendly practices are now the prime concern of the worldly regulatory bodies, and the SDGs are strong advocates of green practices by business firms.

This study further adds to the growing body of knowledge about GC, as well as the significant effects of cognitive processes like GT and GTL. The research adds to our knowledge of GTL and its relevance in promoting green creativity in SMEs. GTL encourages and stimulates followers to achieve the company's environmental goals through green creativity. This research also shows that GTL promotes green thinking, which in turn promotes green innovation. GT is the concept that human cognitive acts and capacities encourage people to take responsibility for environmental issues. The findings also reinforce our hypothesis that GT is a vital link between GTL and GC (Begum et al., 2022). When the firm is strategically oriented toward green innovation, the development of green thinking with the expanding function of GTL will help the firm. Furthermore, the findings of our study add to the growing body of empirical evidence that GTL promotes green creativity. Moreover, the semi-government and government can step forward in developing policies linked to GC that will stimulate the SME sector to establish GTL and GOI, as well as develop environmentally friendly creative work outcomes.

### Theoretical implications

In the context of SMEs, the findings of this study add to the literature on GTL, GOI, and GC. The research presents a model to explain how GTL affects GOI, which in turn affects GC. This research presents numerous particular theoretical contributions based on the findings. First, the findings indicate that cultivating a GOI boosts green creativity. Second, the study confirms the importance of GTL in generating GC in a direct way. Third, in SMEs, GOI serves as a link (mediator) between GTL and GC. Fourth, this research contributes to the body of knowledge on GC by examining its implementation in SMEs. Fifth, by focusing on GTL's interaction with GT and its subsequent impact on green innovation, this study added to the theory. No current study has attempted to determine the influence of GT in mediating the relationship between GTL and GC (Singh et al., 2020; Begum et al., 2022). Sixth, this study contributes to the conceptual model, by empirically analyzing the extent to which green thinking mediates the influence of GTL on GC. Employees who are aware of environmental challenges and use their cognitive ability to generate suggestions for greener production are said to be green thinkers. Individual cognitive responses are critical for generating long-term behaviors that protect the environment from the harm caused by conventional product development and disposal. As a result, we improve our understanding of the important aspects that affect GC by introducing the notion of GT and exploring potential consequences on GC. This research concludes that SMEs can make a substantial contribution to environmental protection by employing a set of creative activities, such as GTL and GC. Additionally, businesses must link the appropriate leadership styles and develop greater levels of GC.

### Implications for managerial practice

The outcomes of this research have practical significance for top managers and management. First, the findings suggest that GTL has a considerable impact on GC. Our findings significantly support the use of GTL to encourage GC in the SMEs of Pakistan. Our findings also show that, in order to improve their market image, managers should include the green concept into their organizational culture and encourage people to think green and generate green concepts and ideas, and approaches and strategies for producing green products and services. Second, our findings imply that in SMEs, green thinking is a key factor in green innovation. Management must understand the significance of incorporating green thinking into their organizational goals. Green thinking may aid in the pursuit of a paradigm change in order to attain long-term sustainability in GC.

This research presented critical recommendations for managers to develop a GOI through green GTL in SMEs. If the firms want to inspire GC among their employees, they will need to incorporate GTL and GOI into their long-term environmental plans. By adopting GTL and a green corporate identity, managers and management can play an essential role in establishing environmentally friendly service behavior. To improve their employees' green creativity, SME managers and management should organize training activities and programs to comprehend eco-friendly approaches that they may use to promote GTL and GOI among their employees. Leaders should work hard to develop the necessary abilities in themselves and their workers in order to create a green corporate identity and make the necessary resources available to achieve GC. Organizational managers may be recommended to train themselves and their employees in order to acquire skills that aid in increasing resource commitment to the firm, so that individuals do not waste too much time, effort, or capital while carrying out those tasks.

Finally, the study's findings show that SMEs in Pakistan, particularly those in Punjab Province, need to recognize their eco-friendly responsibilities toward the planet for the purpose of taking care of a green planet and achieving a higher level of GC. The challenge for these SMEs would be to come up with appropriate leaders and achieve a high degree of GC.

## Limitations and directions for future work

There are certain limitations to this research that need to be highlighted. Since this study is based on SMEs in Pakistan, particularly in the Punjab Province, there is a need to perform additional research in other provinces of Pakistan and in other sectors, such as educational, IT, banking, and health sectors, in order to validate and generalize the findings of this research in the future. Second, when analyzing the relationship between GTL and GC, cultural aspects were not taken into account. The cultural aspect may be considered in future studies. Finally, this study was a cross-sectional study, and future studies may conduct longitudinal studies to gain a better understanding of the complexity among these variables leading toward environmental performance.

## Data availability statement

The raw data supporting the conclusions of this article will be made available by the authors, without undue reservation.

## Ethics statement

Ethical review and approval was not required for the study on human participants in accordance with the local legislation and institutional requirements. The patients/participants provided their written informed consent to participate in this study.

## Author contributions

BA-G: conceptualization, formal analysis, data curation, revising the manuscript, and writing—original draft. HG: conceptualization, funding acquisition, proofreading, and curation. SS and RA: conceptualization, data collection, and writing—original draft. All authors contributed to the article and approved the submitted version.

## Funding

The publication of this article was funded by Qatar National Library.

## Conflict of interest

The authors declare that the research was conducted in the absence of any commercial or financial relationships that could be construed as a potential conflict of interest.

## Publisher's note

All claims expressed in this article are solely those of the authors and do not necessarily represent those of their affiliated organizations, or those of the publisher, the editors and the reviewers. Any product that may be evaluated in this article, or claim that may be made by its manufacturer, is not guaranteed or endorsed by the publisher.

## References

[B1] Ahmeda U. Mozammelb S. Zamanc F. (2020). Green HRM and green innovation: Can green transformational leadership moderate: case of pharmaceutical firms in Australia. Syst. Rev. Pharm. 11, 616–617. 10.31838/srp.2020.7.86

[B2] Albert S. Whetten D. (1985). Organizational identity. Res. Organ. Behav. 7, 263–295.

[B3] Ali F. Ashfaq M. Begum S. Ali A. (2020). How “green” thinking and altruism translate into purchasing intentions for electronics products: the intrinsic-extrinsic motivation mechanism. Sustain. Prod. Consumpt. 24, 281–291. 10.1016/j.spc.2020.07.013

[B4] Amani P. Lindbom I. Sundström B. Östergren K. (2015). Green-lean synergy-root-cause analysis in food waste prevention. Int. J. Food Syst. Dyn. 6, 99–109. 10.22004/ag.econ.208874

[B5] Andriopoulos C. (2001). Determinants of organisational creativity: a literature review. Manage. Dec. 39, 834–840. 10.1108/0025174011040232830859882

[B6] Appelbaum E. Bailey T. Berg P. Kalleberg A. L. Bailey T. A. (2000). Manufacturing Advantage: Why High-Performance Work Systems Pay Off . New York, NY; Tokyo: Cornell University Press.

[B7] Avolio B. J. Bass B. M. Jung D. I. (1999). Re-examining the components of transformational and transactional leadership using the multifactor leadership questionnaire. J. Occup. Organ. Psychol. 72, 441–462. 10.1348/096317999166789

[B8] Awan U. Sroufe R. Kraslawski A. (2019). Creativity enables sustainable development: supplier engagement as a boundary condition for the positive effect on green innovation. J. Clean. Prod. 226, 172–185. 10.1016/j.jclepro.2019.03.308

[B9] Bai C. Kusi-Sarpong S. Sarkis J. (2017). An implementation path for green information technology systems in the Ghanaian mining industry. J. Clean. Prod. 164, 1105–1123. 10.1016/j.jclepro.2017.05.151

[B10] Bass B. M. (1985a). Leadership and Performance Beyond Expectations. New York, NY: Free Press.

[B11] Bass B. M. (1985b). Leadership: good, better, best. Organ. Dyn. 13, 26–40. 10.1016/0090-2616(85)90028-2

[B12] Bass B. M. (1998). Transformational Leadership: Industrial, Military, and Educational Impact. Mahwah, NJ: Erlbaum.

[B13] Bass B. M. (2000). The future of leadership in learning organizations. J. Lead. Stud. 7, 18–40. 10.1177/107179190000700302

[B14] Begum S. Ashfaq M. Xia E. Awan U. (2022). Does green transformational leadership lead to green innovation? The role of green thinking and creative process engagement. Bus. Strat. Environ. 31, 580–597. 10.1002/bse.2911

[B15] Benet-Martínez V. Leu J. Lee F. Morris M. (2002). Negotiating biculturalism: cultural frame switching in biculturals with oppositional versus compatible cultural identities. J. Cross Cult. Psychol. 33, 492–516. 10.1177/0022022102033005005

[B16] Bhutta M. K. S. Khan J. H. Omar A. Asad U. (2009). An exploratory study of the characteristics affecting the success of SMEs in Pakistan. Int. J. Entrepreneurship Small Bus. 7, 107–122. 10.1504/IJESB.2009.021612

[B17] Boxall P. Steeneveld M. (1999). Human resource strategy and competitive advantage: a longitudinal study of engineering consultancies. J. Manage. Stud. 36, 443–463. 10.1111/1467-6486.00144

[B18] Caldera H. T. S. Desha C. Dawes L. (2018). Exploring the characteristics of sustainable business practice in small and medium-sized enterprises: experiences from the Australian manufacturing industry. J. Clean. Prod. 177, 338–349. 10.1016/j.jclepro.2017.12.265

[B19] Caldera H. T. S. Desha C. Dawes L. (2019). Transforming manufacturing to be ‘good for planet and people’, through enabling lean and green thinking in small and medium-sized enterprises. Sustain. Earth 2, 4. 10.1186/s42055-019-0011-z

[B20] Chen Y.-S. (2011). Green organizational identity: sources and consequence. Manage. Dec. 49, 384–404. 10.1108/00251741111120761

[B21] Chen Y. S. (2008). The driver of green innovation and green image - green core competence. J. Bus. Ethics 81, 531–543. 10.1007/s10551-007-9522-1

[B22] Chen Y. S. Chang C. H. (2013). The determinants of green product development performance: green dynamic capabilities, green transformational leadership, and green creativity. J. Bus. Ethics 116, 107–119. 10.1007/s10551-012-1452-x

[B23] Cristina De Stefano M. Montes-Sancho M. J. Busch T. (2016). A natural resource-based view of climate change: innovation challenges in the automobile industry. J. Clean. Prod. 139, 1436–1448. 10.1016/j.jclepro.2016.08.023

[B24] Dhingra R. Kress R. Upreti G. (2014). Does lean mean green?. J. Clean. Prod. 85, 1–7. 10.1016/j.jclepro.2014.10.032

[B25] Ding X. Appolloni A. Shahzad M. (2022). Environmental administrative penalty, corporate environmental disclosures and the cost of debt. J. Clean. Prod. 332, 129919. 10.1016/j.jclepro.2021.129919

[B26] Eide A. E. Saether E. A. Aspelund A. (2020). An investigation of leaders' motivation, intellectual leadership, and sustainability strategy in relation to Norwegian manufacturers' performance. J. Clean. Prod. 254, 120053. 10.1016/j.jclepro.2020.120053

[B27] Elkins T. Keller R. T. (2003). Leadership in research and development organizations: a literature review and conceptual framework. Leadersh. Q. 14, 587–606. 10.1016/S1048-9843(03)00053-5

[B28] Fernandez E. Junquera B. Ordiz M. (2003). Organizational culture and human resources in the environmental issue: a review of the literature. Int. J. Hum. Resourc. Manage. 14, 634–656. 10.1080/0958519032000057628

[B29] Fiol C. M. (1991). Managing culture as a competitive resource: an identity-based view of sustainable competitive advantage. J. Manage. 17, 191–211. 10.1177/014920639101700112

[B30] Gardner W. L. Avolio B. J. (1998). The charismatic relationship: a dramaturgical perspective. Acad. Manage. Rev. 23, 32–58. 10.2307/259098

[B31] Garg S. Dhar R. L. (2014). Effects of stress, LMX and perceived organizational support on service quality: mediating effects of organizational commitment. J. Hosp. Tour. Manage. 21, 64–75. 10.1016/j.jhtm.2014.07.002

[B32] Gebauer H. (2011). Exploring the contribution of management innovation to the evolution of dynamic capabilities. Indust. Market. Manage. 40, 1238–1250. 10.1016/j.indmarman.2011.10.003

[B33] Gong Y. Huang J. C. Farh J. L. (2009). Employee learning orientation, transformational leadership, and employee creativity: the mediating role of employee creative self-efficacy. Acad. Manage. J. 52, 765–778. 10.5465/amj.2009.43670890

[B34] Gumusluoglu L. Ilsev A. (2009). Transformational leadership, creativity, and organizational innovation. J. Bus. Res. 62, 461–473. 10.1016/j.jbusres.2007.07.03235529553

[B35] Gupta V. Singh S. Kumar S. Bhattacharya A. (2012). Linking leadership to employee creativity: a study of indian R&D laboratories. Indian J. Ind. Relat. 48, 120–136. 10.1108/01409171311284594

[B36] Hair J. F. Black W. C. Babin B. J. Anderson R. E. (2010). Multivariate Data Analysis: A Global Perspective, 7th edn. Upper Saddle River, NJ: Pearson Prentice Hall.

[B37] Hair J. F. Jr. Sarstedt M. Hopkins L. Kuppelwieser V. G. (2014). Partial least squares structural equation modeling (PLS-SEM). Euro. Bus. Rev. 26, 106–121. 10.1108/EBR-10-2013-012833203091

[B38] Halbesleben J. R. B. Novicevic M. M. Harvey M. G. Buckley M. R. (2003). Awareness of temporal complexity in leadership of creativity and innovation: a competency-based model. Leadersh. Q. 14, 433–454. 10.1016/S1048-9843(03)00046-8

[B39] Hines P. (2009). Lean and Green. Source Magazine the Home of Lean Thinking, 3rd Edn. Caerphilly: SA Partners.

[B40] IQAir (2021). Air Quality and Pollution City Ranking. World Air Quality. Available online at: https://www.iqair.com/world-air-quality-ranking

[B41] Jia J. Liu H. Chin T. Hu D. (2018). The continuous mediating effects of GHRM on employees' green passion via transformational leadership and green creativity. Sustainability 10, 3237. 10.3390/su10093237

[B42] Jose P. E. (2013). Doing Statistical Mediation & Moderation. London: Guilford.

[B43] Jung D. I. Chow C. Wu A. (2003). The role of transformational leadership in enhancing organizational innovation: hypotheses and some preliminary findings. Leadersh. Q. 14, 525–544. 10.1016/S1048-9843(03)00050-X

[B44] Kaltiainen J. Hakanen J. (2022). Fostering task and adaptive performance through employee well-being: the role of servant leadership. BRQ Bus. Res. Q. 25, 28–43. 10.1177/2340944420981599

[B45] Khan S. A. R. Razzaq A. Yu Z. Miller S. (2021). Industry 4.0 and circular economy practices: a new era business strategies for environmental sustainability. Bus. Strat. Environ. 30, 4001–4014. 10.1002/bse.2853

[B46] Kline R. B. (2011). Principles and Practice of Structural Equation Modeling (3 Baski). New York, NY: Guilford.

[B47] Lee K. (2008). Opportunities for green marketing: young consumers. Market. Intell. Plan. 26, 573–586. 10.1108/02634500810902839

[B48] Li W. Bhutto T. A. Xuhui W. Maitlo Q. Zafar A. U. Bhutto N. A. (2020). Unlocking employees' green creativity: the effects of green transformational leadership, green intrinsic, and extrinsic motivation. J. Clean. Prod. 255, 120229. 10.1016/j.jclepro.2020.120229

[B49] Liu D. Liao H. Loi R. (2012). The dark side of leadership: a three-level investigation of the cascading effect of abusive supervision on employee creativity. Acad. Manage. J. 55, 1187–1212. 10.5465/amj.2010.0400

[B50] Lopez-Cabrales A. DeNisi A. (2021). The road to more sustainable firms in the face of a pandemic: changes needed in employment relationships. BRQ Bus. Res. Q. 24, 241–248. 10.1177/23409444211017913

[B51] Lu H. Xu W. Cai S. Yang F. Chen Q. (2022). Does top management team responsible leadership help employees go green? the role of green human resource management and environmental felt?responsibility. Corp. Soc. Responsib. Environ. Manag. 29, 843–859. 10.1002/csr.2239

[B52] Lusiani M. Abidin Z. Fitrianingsih D. Yusnita E. Adiwinata D. Rachmaniah D.. (2020). Effect of servant, digital and green leadership toward business performance: evidence from Indonesian manufacturing. Syst. Rev. Pharm. 11, 1351–1361. 10.31838/srp.2020.11.192

[B53] Mittal S. Dhar R. L. (2015). Transformational leadership and employee creativity: mediating role of creative self-efficacy and moderating role of knowledge sharing. Manag. Decis. 53, 894–910. 10.1108/MD-07-2014-0464

[B54] Mittal S. Dhar R. L. (2016). Effect of green transformational leadership on green creativity: a study of tourist hotels. Tour. Manag. 57, 118–127. 10.1016/j.tourman.2016.05.007

[B55] Ohno T. Bodek N. (2019). Toyota Production System: Beyond Large-Scale Production. New York, NY; Japan: Productivity Press. 10.4324/9780429273018

[B56] Ojo A. O. Raman M. Downe A. G. (2019). Toward green computing practices: a Malaysian study of green belief and attitude among information technology professionals. J. Clean. Prod. 224, 246–255. 10.1016/j.jclepro.2019.03.237

[B57] Packalen S. (2010). Culture and sustainability. Corp. Soc. Responsib. Environ. Manag. 17, 118–121 10.1002/csr.236

[B58] Pham D. T. Pham P. T. N. Thomas A. (2008). Integrated production machines and systems–beyond lean manufacturing. J. Manufact. Technol. Manage. 19, 695–711. 10.1108/17410380810888094

[B59] Przychodzen W. G_omez-Bezares F. Przychodzen J. (2018). Green information technologies practices and financial performance e the empirical evidence from German publicly traded companies. J. Clean. Prod. 201, 570–579. 10.1016/j.jclepro.2018.08.081

[B60] Rademaker C. A. Royne M. B. (2018). Thinking green: how marketing managers select media for consumer acceptance. J. Bus. Strat. 39, 30–38. 10.1108/JBS-05-2017-0070

[B61] Rehman A. Arif M. S. Tufail M. A. Shahzad S. M. Farooq T. H. Ahmed W.. (2021). Biochar potential to relegate metal toxicity effects is more soil driven than plant system: a global meta-analysis. J. Clean. Prod. 316, 128276. 10.1016/j.jclepro.2021.128276

[B62] Salas-Vallina A. Alegre J. (2018). Unselfish leaders? Understanding the role of altruistic leadership and organizational learning on happiness at work (HAW). Leadersh. Organ. Dev. J. 39, 39–49. 10.1108/LODJ-11-2017-0345

[B63] Sarros J. C. Cooper B. K. Santora J. C. (2008). Building a climate for innovation through transformational leadership and organizational culture. J. Leadersh. Organ. Stud. 15, 145–158. 10.1177/1548051808324100

[B64] SEMDA (2020). COVID-19 SME Liquidity Support And Business Formalization Survey Report. SEMDA.

[B65] Sethi R. (2000). Super ordinate identity in cross-functional product development teams: its antecedents and effect on new product performance. J. Acad. Mark. Sci. 28, 330–344.

[B66] Shah S. H. A. Haider A. Alvi B. Kiani O. I. Arif M. (2021). The impact of leadership styles on turnover intentions directly and through organizational citizenship behavior: small and medium enterprises in Pakistan. Element. Educ. Online. 20, 2752–2773. 10.17051/ilkonline.2021.04.315

[B67] Shah S. H. A. Sultana A. Gul A. Sajjad S. Aziz S. Basit A.. (2020). Transformational leadership influence on innovation directly and indirectly through affective commitment in hotel industry of Malaysia. Int. Rev. Manag. Mar. 10, 22–28.

[B68] Shin S. J. Zhou J. (2003). Transformational leadership, conservation, and creativity: evidence from Korea. Acad. Manage. J. 46, 703–714. 10.5465/30040662

[B69] Singh S. K. Del Giudice M. Chierici R. Graziano D. (2020). Green innovation and environmental performance: The role of green transformational leadership and green human resource management. Tech. Forecast. Soc. Change 150, 119762. 10.1016/j.techfore.2019.119762

[B70] Song W. Yu H. (2017). Green innovation strategy and green innovation: the roles of green creativity and green organizational identity. Corp. Soc. Responsib. Environ. Manag. 25, 135–150. 10.1002/csr.1445

[B71] Sun X. El Askary A. Meo M. S. Zafar N. U. A. Hussain B. (2022). Green transformational leadership and environmental performance in small and medium enterprises. Econ. Res. 1, 1–19. 10.1080/1331677X.2021.202512735939187

[B72] Takeuchi R. Lepak D. P. Wang H. Takeuchi K. (2007). An empirical examination of the mechanisms mediating between high-performance work systems and the performance of Japanese organizations. J. Appl. Psychol. 92, 1069. 10.1037/0021-9010.92.4.1069 17638466

[B73] Thanki S. Govindan K. Thakkar J. (2016). An investigation on lean-green implementation practices in Indian SMEs using analytical hierarchy process (AHP) approach. J. Clean. Prod. 135, 284–298. 10.1016/j.jclepro.2016.06.105

[B74] Tuan L. T. (2019). Environmentally-specific servant leadership and green creativity among tourism employees: dual mediation paths. J. Sustain. Tour. 28, 86–109. 10.1080/09669582.2019.1675674

[B75] Verrier B. Rose B. Caillaud E. Remita H. (2014). Combining organizational performance with sustainable development issues: the lean and green project benchmarking repository. J. Clean. Prod. 85, 83–93. 10.1016/j.jclepro.2013.12.023

[B76] Wang C. J. Tsai H. T. Tsai M. T. (2014). Linking transformational leadership and employee creativity in the hospitality industry: the influences of creative role identity, creative self-efficacy, and job complexity. Tour. Manage. 40, 79–89. 10.1016/j.tourman.2013.05.008

[B77] Wang X. Zhou K. Liu W. (2018). Value congruence: a study of green transformational leadership and employee green behavior. Front. Psychol. 9, 1946. 10.3389/fpsyg.2018.01946 30356727 PMC6189445

[B78] Whetten D. A. (2006). Albert and Whetten revisited: strengthening the concept of organizational identity. J. Manag. Inq. 15, 219–234.

[B79] Woodman R. W. Sawyer J. E. Griffin R. W. (1993). Toward a theory of organizational creativity. Acad. Manage. Rev. 18, 293–321. 10.2307/258761

[B80] Wyer P. Donohoe S. Matthews P. (2010). Fostering strategic learning capability to enhance creativity in small service businesses. Serv. Bus. 4, 9–26. 10.1007/s11628-009-0086-2

[B81] Xie X. Huo J. Zou H. (2019). Green process innovation, green product innovation, and corporate financial performance: a content analysis method. J. Bus. Res. 101, 697–706. 10.1016/j.jbusres.2019.01.010

[B82] Yong J. Y. Yusliza M. Y. Ramayah T. Fawehinmi O. (2019). Nexus between green intellectual capital and green human resource management. J. Clean. Prod. 215, 364–374. 10.1016/j.jclepro.2018.12.306

[B83] Yu W. Chavez R. Feng M. Wong C. Y. Fynes B. (2020). Green human resource management and environmental cooperation: an ability-motivation-opportunity and contingency perspective. Int. J. Prod. Econ. 219, 224–235. 10.1016/j.ijpe.2019.06.013

[B84] Zafar A. Nisar Q. A. Shoukat M. Ikram M. (2017). Green transformational leadership and green performance: the mediating role of green mindfulness and green self-efficacy. Int. J. Manage. Excell. 9, 1059–1066. 10.17722/ijme.v9i2.916

[B85] Zailani S. Govindan K. Iranmanesh M. Shaharudin M. R. Sia Chong Y. (2015). Green innovation adoption in automotive supply chain: the Malaysian case. J. Clean. Prod. 108, 1115–1122. 10.1016/j.jclepro.2015.06.039

